# Use of swabs for dry collection of self-samples to detect human papillomavirus among Malagasy women

**DOI:** 10.1186/s13027-016-0059-8

**Published:** 2016-03-17

**Authors:** Pierre Vassilakos, Rosa Catarino, Stephanie Bougel, Maria Munoz, Caroline Benski, Ulrike Meyer-Hamme, Jeromine Jinoro, Josea Lea Heriniainasolo, Patrick Petignat

**Affiliations:** Geneva Foundation for Medical Education and Research, Route de Ferney 150, 1211 Geneva, Switzerland; Division of Gynaecology, Department of Gynaecology and Obstetrics, Geneva University Hospitals, Boulevard de la Cluse 30, 1206 Geneva, Switzerland; Biopath Lab SA, Rue du Liseron 11, 1006 Lausanne, Switzerland; Saint Damien Healthcare Centre, Ambanja, Madagascar

**Keywords:** Self-sampling, Dry swab, Human papillomavirus (HPV), HPV testing, HPV prevalence, Cervical cancer

## Abstract

**Background:**

Most women in developing countries have never attended cervical screening programmes and often little information exists on type-specific human papillomavirus (HPV) prevalence among these populations. Self-sampling for HPV testing (self-HPV) using a dry swab may be useful for establishing a screening program and evaluating HPV prevalence. Our aim was to evaluate self-HPV using a dry swab stored at room temperature.

**Methods:**

This community-based study in Madagascar consisted of 449 women aged 30–65. Eligible women were provided a dry swab to perform self-HPV. HPV analysis was accomplished by two different real-time PCR tests using the same extracted DNA from the samples.

**Results:**

Overall, 52 (11.6 %) specimens were invalid for HPV detection. The delay between sampling and laboratory processing of DNA extraction considerably increased invalid results. Overall HPV prevalence of 14 hrHPV types detected by the two PCR tests was found to be 38.2 % (*n* = 152). Distribution of 19 hrHPV and 9 low-risk HPV (lrHPV) types revealed most frequently 53 and 68 among hrHPV and HPV 54, HPV 70 and HPV 42 among lrHPV. Agreement between the two PCR methods for any of the 14 high-risk HPV (hrHPV) strains detected was 89.9 % (kappa = 0.77, 95 % CI: 0.71–0.84). In 385 (85.7 %) samples the DNA load of ß-globin demonstrated a signal with medium or high level copies. Conversely, in 28 (60.9 %) invalid samples the signal was undetectable. The HPV-DNA load signal was predominantly of intermediate level (58.5 %, *n* = 218).

**Conclusions:**

Self-HPV using a dry swab stored at room temperature could be a useful method for HPV screening and for conducting population-based surveys on HPV prevalence in resource-poor settings.

## Background

The high sensitivity of clinically validated HPV tests together with the limitations associated with a Pap test [1], led to the recommendation of HPV testing as a replacement of cytology for primary screening in the past decade [2]. Besides these issues, logistic difficulties to introduce cytology-based screening programs in low- and medium-income countries (LMIC) led to the validation of high-risk HPV (hrHPV) testing as an alternative for primary screening by the World Health Organization [3]. However, a screening program is only successful when most of the eligible women participate. In LMIC, many women are unable to visit healthcare facilities, and cervical cancer (CC) screening programs requiring speculum examination are difficult to implement because of logistical issues. A promising alternative for these women is screening by means of self-HPV testing (self-HPV) [4].

Several studies have shown that Self-HPV is acceptable to women [5–7] and compares favourably to clinician-collected specimens [5, 8–10]. A recent meta-analysis showed that when PCR-based HPV tests were used, the clinical performance of self-HPV was similar to clinician-collected samples, irrespective of the type of device used [11].

Self-HPV implementation in LMIC requires a careful analysis of logistics. The choice of HPV test and collection device is of paramount importance. Optimization of transport and storage still remains a challenging issue. Most of the collection devices are stored in specimen transport medium or liquid-based cytology media, which preserve HPV-DNA at room temperature but are expensive and unavailable in a resource-poor context. Additionally, they are toxic and flammable and spillage and leakage can happen during collection and transit.

Compared with liquid media, dry swabs have the advantage of being less expensive and not requiring a special media, so the sample can be safely transported at ambient temperature. Studies demonstrated a good agreement (70–92 %) for HPV detection between dry and wet swabs [12–15].

The goal of this study was to determine, within field conditions of CC screening in Madagascar, the validity of HPV detection in self-obtained samples using a dry swab stored at room temperature. Additionally, we analysed HPV prevalence and genotype distribution among the screened population.

## Methods

### Study setting

The study was conducted by the University of Geneva in collaboration with Madagascar’s Health and Family Planning Ministry and the Saint-Damien Healthcare Centre in Ambanja, a mainly rural area with 125,056 inhabitants.

All participants signed an informed consent form before inclusion. The study was conducted from February to March 2015 in the Saint-Damien Healthcare Centre. Ethical approval of the study was obtained from the Malgasch National Commission for the Ethics of Science and Technology, as well as from the Ethical Cantonal Board of Geneva, Switzerland (CER: 14–071).

### Study participants and procedures

We recruited 449 women aged between 30 and 65 years. Exclusion criteria were former conisation or hysterectomy and pregnancy beyond 20 weeks.

Participants received educational intervention on cancer and HPV infection. It was followed by information on how to use the self-HPV, written in the local dialect language. At the same time the participants received a sterile, cotton-tipped swab in a dry, labelled tube. Self-HPV was always performed without supervision. Then participants were invited to answer a questionnaire regarding socio-demographic information. HPV tests were analysed in Switzerland. HPV test analysis was done in a minimum time of 15 days after the sampling.

HPV-negative women were informed that they were not at risk for CC and that they would not require a test within the next 5 years. HrHPV-positive women were invited for colposcopic examination. A biopsy on acetowhite lesions coupled with endocervical curettage was performed, or a biopsy at the 6 o’clock position and endocervical curettage if colposcopy was normal. Treatment and follow-up was proposed according to the histological diagnosis. In case of an invalid result, women were asked to repeat their self-sampling. Histology was also analysed in Switzerland.

### Laboratory methods

Upon arrival to the laboratory, the swabs were suspended in 4.3 mL of cobas® PCR media and were pulse vortexed for 3 × 10 s. A volume of 400 μL of each sample was used for DNA extraction and the rest of the sample was stored at 4 °C. DNA extraction was carried out using the cobas® HPV Test (Roche Molecular Systems). Nucleic extracts were then stored at −20 °C.

HPV analysis was accomplished by two different real-time PCR tests: the cobas® HPV Test (cobas) and the Anyplex™ II HPV28 (H28) test (Seegene, Seoul, South Korea), using the same extracted DNA from samples.

Amplification and detection were first carried out with cobas (at Biopath Lab, Lausanne), which detects 12 pooled hrHPV genotypes (HPV 31, 33, 35, 39, 45, 51, 52, 56, 58, 59, 66, and 68) and concurrently provides separate results for HPV-16 and HPV-18. The detection is based on amplification of the L1 gene and TaqMan probes [16]. The human reference gene ß-globin s also detected.

Amplification and detection using the stored DNA extracts were then performed with the H28 test using the CFX96™ real-time thermocycler (at Buhlmann Laboratories AG Schönenbuch, Switzerland). Data recording and interpretation were automated. Details of the procedure and evaluation were described by Estrade et al. [17], Kim et al. [18] and Kwon et al. [19].

H28 is a semi-quantitative real-time multiplex PCR assay for screening and HPV genotyping. This test uses Dual Priming Oligonucleotides (DPO™) and Tagging Oligonucleotide Cleavage and Extension (TOCE™) technologies and allows to simultaneously detect 19 high-risk HPVs (including types 16, 18, 26, 31, 33, 35, 39, 45, 51, 52, 53, 56, 58, 59, 66, 68, 69, 73 and 82) and 9 low-risk HPVs (including types 6, 11, 40, 42, 43, 44, 54, 61 and 70). ß-globin is also detected for internal control of assay validity.

Knowledge of the step at which the melting curve becomes positive allows for semi-quantification of the DNA load of ß-globin gene and HPV genomes, from low (+; positive after 40 PCR cycles, <10^2^ copies/reaction), to intermediate (++; positive within 31 to 39 PCR cycles, ≥10^2^ and < 10^5^ copies/reaction), to high (+++; positive before 31 PCR cycles, ≥ 10^5^ copies/reaction) (17).

### Data analysis and statistics

Data were analysed with STATA 13 software package (StataCorp, Texas, USA).

Inter-rater agreement statistics and kappa coefficient with 95 % confidence intervals, percent total agreement and percent positive agreement were calculated for the paired results obtained by the cobas and H28 tests. The calculation was restricted to 14 hrHPV.

The trend of association between HPV results and DNA load was evaluated with the chi-square test. We also evaluated the effect of transport time on sample degradation by applying a Kaplan–Meier failure estimate.

The HPV prevalence was calculated from the number of positive cases divided from the number of tested specimens by both PCR methods. HPV positivity was distributed by age-group.

HPV type distribution in single and multiple infections of 19 hrHPVs and 9 lrHPVs was explored using the H28 test.

## Results

### Sample characteristics

The study included 449 self-obtained swab specimens from women whose median age was 43 (IQR: 36–51). Overall, 52 (11.6 %) specimens were invalid for HPV detection, 44 (9.8 %) were invalid for cobas, 46 (10.0 %) were invalid for H28 and 38 (73.1 %) were concordantly classified as invalid: 6 were invalid by H28 but negative by cobas, 5 were invalid by cobas but negative by H28, 1 was invalid by cobas but positive by H28 and 2 were invalid by H28 but positive by cobas (data not shown).

The overall prevalence for the 14 hrHPV types detected by one of the two methods was 38.2 % (152/397); 19.7 % (30/152) of samples were positive for HPV-16/18, while 88.2 % (134/152) were positive for the pooled 12 hrHPV types. The overall prevalence for 19 hrHPV and 9 lrHPV was 51.8 % (209/403); the prevalence for hrHPV only was 38.6 % (156/403).

Overall, 96 HPV-positive patients came to the colposcopy clinic. There were 6 (6.3 %) cervical intraepithelial neoplasia grade 1 (CIN1), 4 (4.2 %) CIN grade 2 (CIN2), 4 (4.2 %) CIN grade 3 (CIN3) and 2 (2.1 %) squamous cell carcinomas. The overall CIN2 or more severe (CIN2+) prevalence was 10.4 %.

### Agreement between cobas and H28 for HPV-DNA detection by type categories

The agreement was 89.9 % for any HPV type, 97.5 % for HPV-16/18 and 90.7 % for the category with HPV pooled types. The proportion of positive agreement was 84.8, 80.0 and 84.0 % for any hrHPV, HPV-16/18 and pooled HPVs, respectively (Table 1).

**Table 1:** Agreement between cobas and H28 for HPV-DNA detection by type categories

Results cobas/H28	HPV distribution by type categories		
*N* (%)	Any hrHPV^a^	HPV16/18^b^	Pool: 31, 33, 35, 39, 45, 51, 52, 56, 58, 59, 66, 68
Pos/Pos	112	20	97
Pos/Neg	16	5	15
Neg/Pos	24	5	22
Neg/Neg	245	367	263
Total	397	397	397
Kappa (95 % CI)	0.77 (0.71–0.84)	0.79 (0.66–0.92)	0.77 (0.71–0.84)
% Total Agreement	89.9	97.5	90.7
% Positive Agreement^c^	84.8	80.0	84.0
*P* value	<0.001	<0.001	<0.001

*Abbreviations: H28* Seegene Anyplex™ II HPV28, *Pos* Positive, *Neg* Negative, *N* number

^a^Any high-risk HPV: HPV 16 and/or HPV18 and/or HPV 31, 33, 35, 39, 45, 51, 52, 56, 58, 59, 66, 68

^b^HPV16/18: HPV16 and or HPV18

^c^The proportion of positive agreement between paired cobas and H28 samples was calculated using 2a/(f1 + g1), where a is the number of samples that were positive for HPV in both tests, f1 is the number of samples that were positive for cobas, and g1 is the number of samples that were positive for H28

### H28 test results and semi-quantification of ß-globin and HPV-DNA load, stratified on delay between sampling and laboratory processing of DNA extraction (2 to ≥4 weeks)

Overall, in 85.7 % of the samples the DNA load of ß-globin demonstrated a signal with medium- or high-level copies. In 60.9 % of invalid samples the signal was undetectable. For 7 HPV-positive cases the ß-globin was undetectable (Table 2). There were no significant differences in the number of invalid samples nor in DNA load of ß-globin between pre- (≤50 years) and postmenopausal (>50 years) women (*p* = 0.834 and *p* = 0.290, respectively).

**Table 2:** H28 HPV results and semi-quantification of ß-globin DNA load, stratified on delay between sampling and laboratory processing of DNA extraction (2 to ≥4 weeks)

HPV results	ß-globin DNA load^a^					*p* value
	Undetectable	+	++	+++	Total	
	*N* (%)	*N* (%)	*N* (%)	*N* (%)	*N* (%)	
2 weeks						
Positive	1 (2.3)	1 (2.3)	32 (74.4)	9 (20.9)	43 (100.0)	<0.001
High-risk^b^	0	1 (3.3)	23 (76.7)	6 (20.0)	30 (100.0)	
Low-risk^c^	1 (4.6)	0	17 (77.3)	4 (18.2)	22 (100.0)	
Negative	0	0	26 (65.0)	14 (35.0)	40 (100.0)	
Invalid	3 (60.0)	2 (40.0)	0	0	5 (100.0)	
3 weeks						
Positive	2 (3.5)	4 (7.0)	38 (66.7)	13 (22.8)	57 (100.0)	<0.001
High-risk^b^	1 (2.3)	4 (9.1)	30 (68.2)	9 (20.5)	44 (100.0)	
Low-risk^c^	1 (3.9)	2 (7.7)	18 (69.2)	5 (19.2)	26 (100.0)	
Negative	0	0	50 (72.5)	19 (27.5)	69 (100.0)	
Invalid	5 (38.5)	8 (61.5)	0	0	13 (100.0)	
≥4 weeks						<0.001
Positive	4 (3.7)	6 (5.5)	79 (72.5)	20 (18.4)	109 (100.0)	
High-risk^b^	2 (2.4)	5 (6.1)	57 (69.5)	18 (22.0)	82 (100.0)	
Low-risk^c^	3 (4.6)	3 (4.6)	51 (78.5)	8 (12.3)	65 (100.0)	
Negative	0	0	62 (72.9)	23 (27.1)	85 (100.0)	
Invalid	20 (71.4)	8 (28.6)	0	0	28 (100.0)	
TOTAL	35 (7.8)	29 (6.5)	287 (63.9)	98 (21.8)	449 (100.0)	

*Abbreviations: N* number

^a^Undetectable, absence of signal; + (low), positive >40 PCR cycles, <10^2^ copies/reaction; ++ (medium) positive within 31 to 39 PCR cycles, ≥10^2^ and < 10^5^ copies/reaction; +++ (high) positive before 31 PCR cycles, ≥ 10^5^ copies/reaction

^b^Positivity for a high-risk HPV type (16, 18, 26, 31, 33, 35, 39, 45, 51, 52, 53, 56, 58, 59, 66, 68, 69, 73 and 82)

^c^Positivity for a low-risk type (6, 11, 40, 42, 43, 44, 54, 61 and 70)

NB For multiple infections positive results were pooled and a single result was generated

**Fig. 1:**
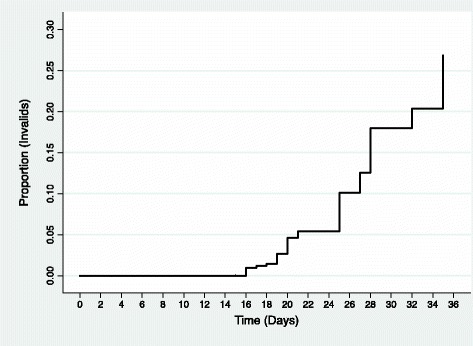
Kaplan-Meier failure estimate showing the proportion of H28 invalid HPV tests over the days. HPV test analysis was done in a minimum time of 15 days following the sampling

The delay between sampling and laboratory processing increased invalid results from 5.7 % after 2 weeks to 9.4 % after 3 weeks and 12.6 % after ≥4 weeks (*p* = 0.177). The mean delay between sampling and laboratory processing was 22.7 ± 0.35 days for valid samples and 24.3 days for invalid samples (*p* = 0.129). In Fig. 1, a Kaplan–Meier failure estimate showing a clear increase in the proportion of invalid tests with time.

Analysis of the relationship between HPV positive test results and DNA load (Table 3) showed that the strength of the load signal was predominantly intermediate (58.5 %). The delay between sampling and DNA extraction did not interfere on the strength of the signal (*p* = 0.206). The strength of the HPV-DNA load was not related to the strength of the ß-globin DNA load (*p* = 0.154). There were no significant differences in HPV-DNA load between pre- and postmenopausal women (*p* = 0.806) (data not shown).

**Table 3:** H28 HPV positive results and semi-quantification of HPV-DNA load, stratified on delay between sampling and laboratory processing of DNA extraction (2 to ≥4 weeks)

HPV positive results	HPV-DNA load^a^				*p* value
	+	++	+++	Total	
	*N* (%)	*N* (%)	*N* (%)	*N* (%)	
2 weeks					
High-risk^b^	20 (44.4)	25 (55.7)	0	45 (100.0)	0.009
Low-risk^c^	9 (34.6)	12 (46.2)	5 (19.2)	26 (100.0)	0.009
3 weeks					
High-risk^b^	22 (37.9)	30 (51.7)	6 (10.3)	58 (100.0)	0.919
Low-risk^c^	11 (34.4)	18 (56.3)	3 (9.4)	32 (100.0)	0.919
≥4 weeks					
High-risk^b^	38 (30.4)	75 (60.0)	12 (9.6)	125 (100.0)	0.409
Low-risk^c^	20 (21.0)	58 (66.7)	9 (10.3)	87 (100.0)	0.491
TOTAL	120 (32.2)	218 (58.5)	35 (9.4)	373 (100.0)	

*Abbreviations: N* number

^a^ + (low), positive >40 PCR cycles, <10^2^ copies/reaction; ++ (medium) positive within 31 to 39 PCR cycles, ≥10^2^ and < 10^5^ copies/reaction; +++ (high) positive before 31 PCR cycles, ≥ 10^5^ copies/reaction

^b^Positivity for a high-risk HPV type (16, 18, 26, 31, 33, 35, 39, 45, 51, 52, 53, 56, 58, 59, 66, 68, 69, 73 and 82)

^c^Positivity for a low-risk type (6, 11, 40, 42, 43, 44, 54, 61 and 70)

NB: For multiple infections each single positive result was counted for high- and low-risk types

### Age-specific HPV prevalence according to cobas and H28 real-time PCR tests

**Fig. 2:**
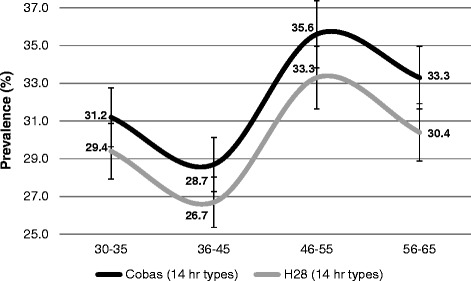
Age-specific HPV prevalence (%) according to cobas and H28 real-time PCR tests. Abbreviations: H28 = Seegene Anyplex II HPV28; hr = high-risk; NB: For multiple infections, results were pooled and a single positive result was generated

The overall prevalence for 14 hrHPV types was found to be 32.1 % with cobas and 29.5 % with H28. Stratification by age demonstrates a decrease from the age group of 30–35 years to the age group 36–45 years. Then a rebound is observed in the age group of 46–55 years, followed by a decrease in the age group of 56–65 years (Fig. 2).

### Type-specific prevalence in mono- and multi-infections according to H28 real-time PCR test (Table 4)

**Table 4:** Type-specific prevalence in mono and multi-infections according to the H28 real-time PCR test

HPV type distribution	Mono-infections	Multi-infections	Total	*p* value *
	*N* (%)	*N* (%)	*N* (%)	
High-risk types (HR)				0.277
16	3 (21.4)	11 (78.6)	14 (6.1)	
18	6 (46.2)	7 (53.9)	13 (5.7)	
26	1 (50.0)	1 (50.0)	2 (0.9)	
31	6 (46.2)	7 (53.9)	13 (5.7)	
33	7 (50.0)	7 (50.0)	14 (6.1)	
35	5 (33.3)	10 (66.7)	15 (6.6)	
39	1 (50.0)	1 (50.0)	2 (0.9)	
45	3 (27.3)	8 (72.7)	11 (4.8)	
51	4 (44.4)	5 (55.6)	9 (4.0)	
52	4 (22.2)	14 (77.8)	18 (7.9)	
53	5 (20.8)	19 (79.2)	24 (10.5)	
56	5 (38.5)	8 (61.5)	13 (5.7)	
58	4 (57.1)	3 (42.9)	7 (3.1)	
59	5 (50.0)	5 (50.0)	10 (4.4)	
66	2 (16.7)	10 (83.3)	12 (5.3)	
68	8 (38.1)	13 (61.9)	21 (9.2)	
69	0	2 (100.0)	2 (0.9)	
73	2 (10.5)	17 (89.5)	19 (8.3)	
82	1 (11.1)	8 (88.9)	9 (4.0)	
All	72 (31.6)	156 (68.4)	228 (100.0)	
Low-risk types (LR)				0.202
6	6 (66.7)	3 (33.3)	9 (6.2)	
11	0	2 (100.0)	2 (1.4)	
40	2 (13.3)	13 (86.7)	15 (10.3)	
42	10 (40.0)	15 (60.0)	25 (17.2)	
43	4 (30.8)	9 (69.2)	13 (9.0)	
44	7 (35.0)	13 (65.0)	20 (13.8)	
54	6 (21.4)	22 (78.6)	28 (19.3)	
61	2 (25.0)	6 (75.0)	8 (5.5)	
70	9 (36.0)	16 (64.0)	25 (17.2)	
All	46 (31.7)	99 (68.3)	145 (100.0)	
TOTAL (HR and LR)	118 (31.6)	255 (68.4)	373 (100)	

*p value Mono-infections vs. Multi-infections

Overall, 31.6 % of the HPV infections were single whereas 68.4 % were multiple. Sixty-one percent of women’s infections revealed hrHPV, particularly HPV-53 and 68, followed by HPV-73, HPV-52, HPV-35, HPV-16, HPV-33, HPV-31 and HPV-18 in decreasing order. Infections with low risk types disclosed particularly HPV-54, HPV-70 and HPV-42, followed by HPV-44, HPV-40 and HPV-6 in decreasing order. There were no significant differences in the distribution of mono-infections vs. multi-infections for hr- and lrHPVs (*p* = 0.300 and *p* = 0.202, respectively).

## Discussion

In Madagascar, besides the lack of effective CC screening, population-based surveys on HPV prevalence are not yet available [20]. In view of these considerations, this study was designed to assess the validity of self-HPV using dry swabs.

Our results indicate that dry swabs provided sufficient amounts of biological material and stable DNA for hrHPV detection by two PCR-based tests (cobas and H28). Agreement between the two real-time PCR assays for the detection of any HPV among the 14 hrHPV types was 89.9 % (kappa = 0.77), despite different technologies and cut-off algorithms. A similarly-strong agreement was observed after the hierarchical categorization of hrHPV types into two groups, according to cancer risk (HPV-16/18 and pool of others).

We investigated the quality of the samples by analysing the relationship between HPV test results and the DNA load. The human reference gene ß-globin, a marker for cellularity, and the HPV-DNA for 19 hrHPV and 9 lrHPV were semi-quantified. Our findings are in line with other studies indicating that the quality of a dry specimen is sufficient for HPV detection [12] but the delay between sampling and HPV detection may interfere in the successful amplification of the reference gene for internal control, generating invalid results [21, 22]. The failure to detect DNA load over time is probably because of genomic DNA degradation in some samples [21]. Baay et al. [22] compared ß-globin concentrations and purity in vaginal samples self-collected by college students on-site to samples that were collected at home and mailed to the study laboratory with a delay ranging from 1 to 23 days (mean 4 days). They observed that DNA yield decreased with longer transport time; however, this had only a minimal effect on PCR amplification. Lin et al. [23], using a referral population, evaluated the stability of samples collected with dry swabs and tested with the cobas 4800 HPV test. They found that sample stored at an ambient, uncontrolled temperature can last up to one month without loss of sensitivity and specificity for detecting high-grade cervical intraepithelial lesion or cancer. Our findings confirm that stability of DNA in the sample collected with a dry swab is maintained, however, specimens processed after 2 weeks were more likely to be invalid for analysis. Concerning the strength of the HPV-DNA load, no detectable impact of the transport time was found on the signal strength (*p* = 0.206) and the strength of HPV-DNA load was not related to the strength of the ß-globin DNA load (*p* = 0.154). Interestingly, in 7 samples with undetectable ß-globin, PCR analysis with H28 revealed positive HPV-DNA. This finding may corroborate the hypothesis that HPV-DNA present predominantly as episomal copies in dry samples is less affected from degradation [21].

Our data show that menopausal status does not affect the quality of samples. Nevertheless, it should be noted that besides DNA degradation because of the transportation time, undetectable DNA may also be the result of an inadequate sampling or the presence of PCR inhibitors [24].

This study provides for the first time information on age-specific HPV prevalence in screened women in Madagascar. Cobas and H28 were in agreement for the overall prevalence rates found for 14 hrHPV types. The pattern of HPV prevalence for the restricted age-range of our study appeared similar to some surveyed African countries, but different to others [25]. The observed peak among women aged 45 years and older may be explained by newly acquired infections or by reactivation of latent HPV infections [26].

The high HPV prevalence found among the screened Malagasy women is consistent with published meta-analyses for Eastern Africa. Systematic reviews reported a HPV prevalence of 33.6 % [25] and 42.2 % [27] in women with normal cytology. The comparison of our data on HPV type distribution with other African countries [27, 28], indicates that the epidemiology of HPV infection is different in the study area. Differences in age pattern of HPV prevalence and type distribution in countries and regions may be related to different sexual habits and migrations of people [29].

In this study we used simple cotton swab for sample collection. Flocked swabs were found to have higher sensitivity to detect HPV infection compared to Dacron swabs [30], which was explained by its higher capacity of adhesion leading to a better proportion of DNA. However, in more recent studies [31, 32], no difference was found between the two swabs types for DNA retrieval.

We do recognize that the approach for HPV testing and subsequent follow-up applied in this study is unrealistic for a routine CC screening program in Madagascar. Besides invalid samples, a long delay between screening and reporting the results may lead to increased dropout of HPV-positive women who need to be recalled for management. A solution is the use of emergent rapid PCR-based methods for detecting HPV-DNA, with minimal requirements for laboratory equipment, enabling primary screening and treatment in a single visit. Another limitation in our analysis is the lack of histopathological results for all tested women, which would be useful as gold-standard for both PCR methods.

Because estimates of HPV prevalence and type-specific distribution in Madagascar are necessary for orienting CC prevention and monitoring the impact of vaccination, our study needs to be completed with estimates of a wider age range (15–60 years).

## Conclusions

In conclusion, our study provides evidence that self-HPV with dry swabs stored at room temperature is a valid alternative for screening with HPV testing in low resource settings or remote areas, though HPV analysis should be performed ideally within 2 weeks. Moreover, our findings suggest that unsupervised self-sampling with dry swabs in conjunction with a PCR-based method is feasible in conducting population-based surveys on HPV prevalence, which are limited or missing in many LMIC.

### Ethical approval

Ethical approval of the study was obtained from the Malgasch National Commission for the Ethics of Science and Technology, as well as from the Ethical Cantonal Board of Geneva, Switzerland (CCER, CER: 14–071).

## Abbreviations

CC: cervical cancer
HPV: human papillomavirus
hrHPV: high-risk HPV
LMIC: low- and medium-income countries
lrHPV: low-risk HPV
self-HPV: self-sampling for HPV testing
